# New Appliances and Things Medical

**Published:** 1898-10-22

**Authors:** 


### NEW APPLIANCES AND THINGS MEDICAL.

[We shall be glad to receive, at our Office, 28 & 29, Southampton Street, Strand, London, W.C., from the manufacturers, specimens of all new preparations and appliances which may be brought out from time to time.]

### PATTISONS' OLD HIGHLAND WHISKEY.

(Leith and London.)

We have received a sample of the above whiskey from the well-known distillers, Messrs. Pattison. The sample before us is of particularly pleasant flavour and of mellow properties, evidently well matured and of fine quality. The bottle is carefully encased in wire netting and sealed with the firm's seal, rendering any tampering with the contents by retailers impossible. It is a whiskey that may be safely recommended by medical men to their patients.

### HARTMANN'S PATENT WOOD-WOOL WADDING AND TISSUE.

(The Sanitary Wood-Wool Company, 26, Thavie's Inn, Holborn Circus, E.C.)

The dressings known by the above descriptive title have been submitted to us and fully tested by practical trial. We would specially call attention to the ready way in which these dressings take up heavy and copious secretions. This is due to the coarseness of the fibre, and the free passage of fluids through the interspaces. The coarse, soft, and pliable strands of the wool fibre are intermingled with and held together by a certain proportion of fine cotton absorbent wool (20 per cent.) Many so-called wood-wool dressings now on the market contain no wood-fibre at all, and answer but indifferently the purposes for which they are intended. The presence of wood-wool fibre can be detected by dipping into aniline chloride, which is readily taken up by ligneous tissues and deeply stained yellow. We have before us a sample of Hartmann's wood-wool tissues tested in this fashion, and in which the woody fibre deeply dyed manifests itself to the obvious extent of about 80 per cent.

### TURTLE SOUP AND CONSOMMÉ DE VOLAILLE.

(V. Benoist's, 36, Piccadilly, W.)

The above excellent examples of invalid specialities have been submitted to our notice by V. Benoist, of Piccadilly. Both of them are preserved in glass bottles, carefully stoppered, and free from contamination by lead or tin. The turtle soup, though of the finest quality and containing a generous proportion of pieces of real turtle, is not unduly rich, or over-seasoned. Owing to its high nutritive properties, careful preparation, and delicacy of flavour, turtle soup should be specially valuable to invalids who require a stimulus to their appetite and good nutritive return for the food swallowed. The consommé is a fine example of a clear chicken soup—appetising, clean to the palate, and nutritious

